# A theoretical investigation on reciprocity-inspired wide-angle spectrally-selective THz absorbers augmented by anisotropic metamaterials

**DOI:** 10.1038/s41598-020-67399-3

**Published:** 2020-06-25

**Authors:** Mansoureh Mohammadi, Hamid Rajabalipanah, Ali Abdolali

**Affiliations:** 1 Department of Electrical Engineering, Iran University of Science and Technology, Tehran, 1684613114 Iran; 2 Applied Electromagnetic Laboratory, School of Electrical Engineering, Iran University of Science and Technology, Tehran, 1684613114 Iran

**Keywords:** Electrical and electronic engineering, Applied physics

## Abstract

In this paper, a theoretical framework relying on the reciprocity theorem is proposed to accurately design a spectrally-selective THz superstrate-loaded metamaterial absorber (SLMA) exhibiting wide-angle feature. By leveraging high-order Floquet harmonics in a generalized transmission line model characterizing the conventional metamaterial absorbers (MAs), it is demonstrated that MAs suffer from impedance mismatch, especially at near grazing angles. From an impedance matching viewpoint, this major challenge is tackled in this paper via two different designs, exploiting a magneto-electric anisotropic Huygens' metamaterial and a multilayer dielectric structure at a certain distance over the MA plane. The numerical results corroborate well the theoretical predictions, elucidating that the proposed SLMA significantly broadens the angular performance of the MA up to near grazing angles (about 80°), where high absorptivity is still achieved in both principal planes. The deteriorating effect of diffraction modes has been comprehensively analyzed. In comparison to the previous wide-angle MA reports based on intricate particle geometries and brute-force optimizations, the proposed design features a straightforward semi-analytical algorithm, which can also be re-developed for microwave, mid-infrared, and optical frequency bands and for any type of MA element. The proposed SLMA would be very promising for various wavelength-selective applications such as sensors and imaging.

## Introduction

A spectrally-selective electromagnetic (EM) absorber is an engineered platform whereby all incident energy is trapped and absorbed at a certain spectral spot, while all other reflection, transmission, and scattering channels are disabled. In the wave of the development of metamaterials (MMs) in almost every technologically relevant spectral range from microwave to the visible, researchers have explored different privileges and properties that MMs may provide in designing spectrally-selective absorbers^1,2^. Landy et al. demonstrated the first perfect metamaterial absorber (MA) composed of a metallic electric resonant split ring and a cut wire separated by a dielectric layer, exhibiting the measured absorptivity of about 88%^3^. The idea of perfect EM absorption is to block the transmission of wave and minimize reflectivity by simultaneously providing impedance matching and lossy host materials^4^. Since then, numerous studies have explored MAs in various pragmatic applications such as security imaging^5^, biomedical detection^6^, and energy harvesting^7^. The majority of these works, however, have mainly been centralized on the target of limited frequency regions; instead, limited reports have been systematically presented to design MAs at terahertz (THz) spectrum^8^. Achieving broadband ^9–12^, perfect/tunable^13^, multiband^14^, polarization-insensitive^15^, and wide-angle^16–18^ absorbing features were the main objectives of previous studies.

Generally speaking, the absorption efficiency of MAs highly depends on the polarization state and the angle of illuminations. Regarding the existing ambiguity in the direction of the incident waves in a multitude of practical scenarios, the development of wide-angle THz MAs is now a subject of major interest^19–22^. The polarization property of the MA is solely dependent on the rotational symmetry of the constituent units^23,24^. Acquiring perfect absorption with the angle-insensitive privilege, however, is a much more daunting task^19^ due to the wave-MA impedance mismatch appearing near the grazing angle where the reflectance quickly approaches 100%. Thus, at extreme angles, matching with conventional methods becomes difficult if not impossible, so that for existing wide-angle MAs, the region close to the end-fire direction is difficult to cover. Recently, an enormous amount of effort has been made to enhance the angular features of MAs with engineered geometries, e.g. split-ring-cross resonator^25^, four-fold rotational symmetric electric resonator with a cross-printed bottom^26^, deep subwavelength unit cell in a multilayer^27^, surrounding via array^28^, and circular sector^29^. Although the sensitivity to the polarization and angle of incidences is reduced to some extent, the vast majority of reports in all spectra deal with sophisticated, optimization-based, and costly designs, as they require the deformation of all array elements^30–37^. Further, these approaches only support certain types of array elements, probably creating features which make them difficult for applications over a large area^38^. For instance, based on an anisotropic perfectly impedance-matched negative-index material, a metamaterial-based approach was described^39^ for making a wide-angle absorber of infrared radiation. Nevertheless, due to the single layer nature of the absorber, it operated only for s-polarized (single-polarization) oblique incidences up to 45°. Besides, via the presented approach, a lossy anisotropic metamaterial must be designed to expose specified real and imaginary parts of the permittivity and permeability at the operating frequency and, thus, the metamaterial design would be very complex. Shen et al*.*^40^ reported a compact single unit cell consisting of three nested electric closed-ring resonators as a microwave triple-band absorber. However, as reported by them, the designed absorber was valid to a wide range of incident angles up to only 50° for both TE and TM polarizations. Meanwhile, the design geometry was selected heuristically, and no exact analysis was presented to formulate (or at least justify) the wide-angle performance of the designed MA. Finally, it worked at the microwave frequency band, not THz spectrum. A simple metamaterial-based wide-angle plasmonic absorber was introduced, fabricated, and experimentally characterized^41^ using angle-resolved infrared spectroscopy. The main focus of that paper was not on expanding the angular operating range of the absorber, but on low-cost designing of a plasmonic absorber via nano-imprint lithography for manufacturing large-area samples and so, the angular performance of the absorber was not high enough for diverse applications. More importantly, the design parameters were obtained based on numerical optimizations, and the semi-analytical formulas given in that manuscript were only provided for several justifications about the impedance matching. There are also other studies that have addressed wide-angle metamaterial absorbers at different parts of the spectrum^42–44^. Nevertheless, in most of them, the design of absorbing metamaterial unit cells relied on trial and error steps, no rigorous theoretical formulation supported the study, and the design was based on a heuristic approach. Another possible solution to realize artificial magnetic response is to use a high-impedance surface (like mushroom structure)^45,46^. Most of these designs are for the microwave frequency range, where each patch is connected to the ground plane with a thin conducting via, forming a “mushroom”. The presence of vias is important for the operation at oblique incidence, where they become useful also in absorber applications. Olli Luukkonen et al*.*^45^ designed a mushroom-like absorber with a high-permittivity substrate (obviously with high-cost fabrication) whose absorption efficiency remained high for incidence wave angles up to 60° at microwave frequencies. In the case of a low-permittivity substrate (logical manufacturing cost), the designed absorber failed to achieve satisfactory results for TE polarization at 60°^45^. Consequently, there is a critical need to develop a new architecture revealing wide-angle THz absorptivity combined with simplicity and feasibility. However, little attention has been paid to discussions about the theories or mechanisms of wide-angle absorption, which is very crucial for potential research on MAs.

Here, a combined version of the wide-angle matching layer in phased-array antennas^47^, and reciprocity theorem^48^ from classical electromagnetics is theoretically established to address a spectrally selective semi-omnidirectional THz absorber. We will serve magneto-electric anisotropy as an additional degree of freedom to provide the required wave-absorber impedance matching at near grazing angles. In a more simple design with respect to the perfect match layers, we require a loss-less anisotropic metamaterial exhibiting only the pre-determined real parts of the permittivity and permeability at the operating frequency. Indeed, we present two matching superstrate layers with uniaxial tensors located at a certain distance from a pre-designed MA, a simple configuration physically accomplishable via Huygens' metamaterial or multilayer dielectrics. For demonstration purposes, the designed wide-angle matching layer is located at a certain distance from a printed-dipole MA to provide minimum reflection levels for a wide angular range of oblique illuminations. We will demonstrate that, with the appropriate selection of the transversal and longitudinal anisotropic parameters characterizing the superstrate layer, strong absorptivity can be acquired at 2 THz upon illumination by a wide range of incident angles (up to 80°). The superstrate synthesis process is aided by a parameter extraction technique, accurately extracting the effective uniaxial components. The proposed superstrate-loaded MA (SLMA) does not require a complex design, and more importantly, can be applied to any type of MA and sensor element.

## Results

### Analytical scheme

**Figure 1:**
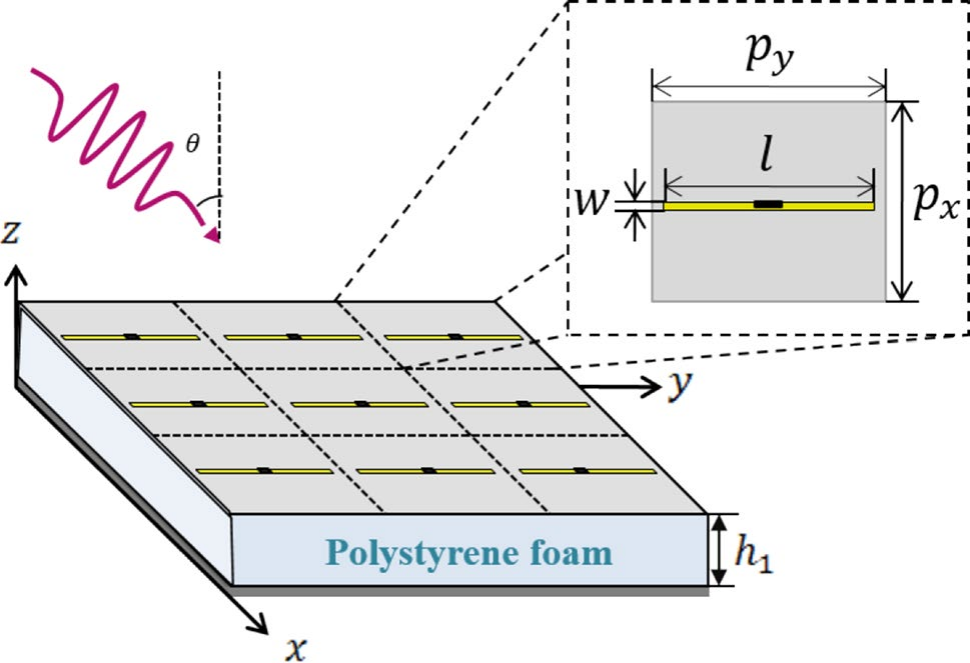
Geometrical model of the THz MA comprising of a planar array of AgNw-loaded dipoles as the basic building block of the proposed SLMA.

In this section, without loss of generality, we will exemplify the proposed design method by leveraging an infinite array of spectrally-selective dipoles as the basic building block of the MA. With the expansion of printed technologies, dipole arrays are known as cost-effective, lightweight, and low-profile meta-devices^49^, so that the advantages of planar MAs becomes much more tangible when employing dipole elements. Furthermore, the use of dipole elements allows an accurate analytical solution for predicting the THz response of MA augmented by auxiliary anisotropic layers. Figure 1 indicates the periodic MA array comprising of zero-thickness dipoles with the length of *l*, width of $w$, and periodicity of $p_{x}$ and $p_{y}$ along x- and y-directions, respectively. The array elements are located on a polystyrene foam^50^ (n_d_ = 1) with the thickness of $h_{1}$ terminated by a gold ground plane ($\sigma = 4.56 \times 10^{7} \;{\text{s/m}}$) to block the transmission. A finite-size ultra-thin sheet of random silver nanowire (AgNw) network^51^ responsible for dissipating the captured energy terminates the middle of each dipole element. The AgNw layer can be thought of as a resistive load for the array of dipoles, which is characterized by its surface resistivity, R_s_ (Ω/m^2^). In a strict sense, the equivalent lumped resistance of the lossy AgNw depends on the unit cell shape such that the scattering surface area per unit is larger in the uniform sheet model than the physical scatterer. As an acceptable estimate^52^, the addition of lossy AgNw layer can be circuitally modelled through a termination resistance of $R_{L} = R_{s} p_{x}^{2} /S$ wherein *S* refers to the surface of the lossy layer along the direction of the induced current. Thanks to the reciprocity theorem, the array of dipoles with the best transmission specifications in the radiating mode will similarly exhibit the best absorption performance when it is suitably terminated in the receiving mode^53^. Consequently, to determine the angular-dependency of the reflectivity for dipole elements in the absorbing mode, one can utilize the scan impedance of the dipoles in the transmitting mode which is defined as the observed impedance when looking into the terminals of a driven dipole element surrounded by an infinite array of similarly driven elements^54^. In this case, the periodic modality of the array allows us to describe the near-field interactions of the dipole array via the Fourier series expansion of the radiating fields. In this way, the TM-polarized fields can be expressed as the superposition of the fields radiated by the y-directed dipole surface currents and the image fields caused by the ground plane in plane waves indexed by different harmonics

1a $$E_{y} \left( {x,y,z} \right) = \sum\limits_{m} {\sum\limits_{n} {E_{x,mn} exp\left( { - jk_{x,m} x} \right) \times exp\left( { - jk_{y,n} y} \right)exp\left( { - j\nu_{mn} z} \right)} }$$

1b $$H_{x} \left( {x,y,z} \right) = \frac{j}{\eta }\sum\limits_{m} {\sum\limits_{n} {\frac{k}{{k_{yn}^{2} - k^{2} }}\left( { - j\nu_{mn} } \right)E_{x,mn} exp\left( { - jk_{x,m} x} \right) \times exp\left( { - jk_{y,n} y} \right)exp\left( { - j\nu_{mn} z} \right)} }$$

in which, $\eta$ and $k$ refer to the characteristic impedance and the wavenumber of the host medium, respectively; and $k_{xm} = k\sin \theta \cos \varphi + 2m\pi /p_{x}$, $k_{yn} = k\sin \theta \sin \varphi + 2n\pi /p_{y}$; and

2 $$\nu_{mn} = \left\{ {\begin{array}{*{20}l} {\sqrt {k^{2} - k_{xm}^{2} - k_{yn}^{2} } } \hfill & {k_{xm}^{2} + k_{yn}^{2} \le k^{2} } \hfill \\ { - j\sqrt {k_{xm}^{2} + k_{yn}^{2} - k^{2} } } \hfill & {k_{xm}^{2} + k_{yn}^{2} > k^{2} } \hfill \\ \end{array} } \right.$$

The surface current density of each unit cell is zero except on the dipole element, which, in turn, can be described by a complex Fourier series. Assuming a sinusoidal loop of current with the maximum (I_0_) at the dipole center, $K_{mn}$ coefficients can be subsequently disclosed using the orthogonality principle^4^

3 $$K_{y} = \mathop \sum \limits_{m = - \infty }^{\infty } \mathop \sum \limits_{n = - \infty }^{\infty } K_{mn} exp\left( { - jk_{x,m} x} \right) \times exp\left( { - jk_{y,n} y} \right)$$

4 $$K_{mn} = \frac{2}{\pi }\frac{{I_{0} }}{{p_{x} }}\frac{l}{{p_{y} }}\frac{{{\sin}\left( {k_{x,m} w/2} \right)}}{{k_{x,m} w/2}}\frac{{{\cos}\left( {k_{y,n} l/2} \right)}}{{1 - \left( {k_{y,n} l/\pi } \right)^{2} }}$$

On the other hand, the scan impedance of each radiating dipole is calculated by

5 $$Z_{a} = \frac{{\int\int { - \frac{{\overline{E} \cdot \overline{K}^{*} }}{2}dS_{unit cell} }}}{{\frac{1}{2}\left| {I_{0} } \right|^{2} }}$$

which yields

6 $$Z_{a} = \frac{2\eta }{{\pi^{2} }}\frac{{l^{2} }}{{p_{x} p_{y} }}\mathop \sum \limits_{m = - \infty }^{\infty } \mathop \sum \limits_{n = - \infty }^{\infty } F_{m}^{2} G_{n}^{2} H_{mn} \left( {1 - exp\left( { - j2 \nu_{mn} h_{1} } \right)} \right)$$

**Figure 2:**
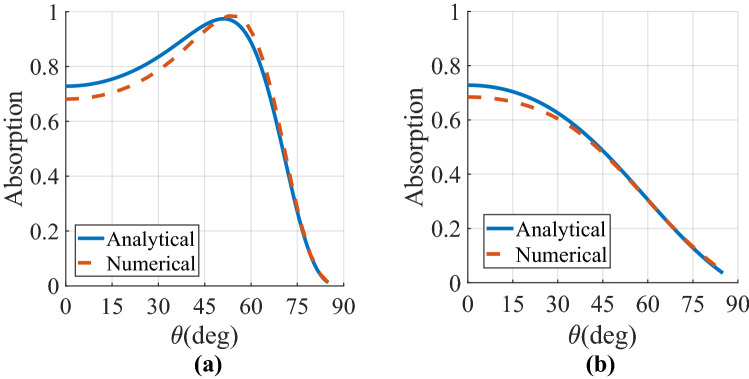
Comparison between the analytical and numerical absorptivity at the specular angle of the MA array of Fig. 1 for incident angles θ ranging from 0° to 90° in (**a**) $\varphi = 0$ and (**b**) $\varphi = 90$ planes. The corresponding parameters of the designed MA are p_x_ = p_y_ = 0.5λ, *l* = 0.48λ, w = 0.02λ, h_1_ = 0.25λ, and R_L_ = 50 Ω, where λ denotes the operating wavelength corresponding to f = 2 THz.

The above sum can be immediately interpreted as the contribution of the propagating/non-propagating Floquet harmonics indexed by m and n to the input impedance of the dipole array. Here, the last term represents the additional contribution of the reflections off the ground plane and^55^

7a $$F_{m} = \frac{{sin\left[ {\frac{\pi w}{\lambda }\left( {\cos \theta_{x} + \frac{m\lambda }{{p_{x} }}} \right)} \right]}}{{\frac{\pi w}{\lambda }\left( {\cos \theta_{x} + \frac{m\lambda }{{p_{x} }}} \right)}}$$

7b $$G_{n} = \frac{{cos\left[ {\frac{\pi }{2}\left( {\cos \theta_{y} + \frac{n\lambda }{{p_{y} }}} \right)} \right]}}{{1 - \left[ {\cos \theta_{y} + \frac{n\lambda }{{p_{y} }}} \right]^{2} }}$$

7c $$H_{mn} = \frac{{\left[ {\cos \theta_{y} + \frac{n\lambda }{{p_{y} }}} \right]^{2} - 1}}{{\sqrt {\left( {\cos \theta_{y} + \frac{n\lambda }{{p_{y} }}} \right)^{2} + \left( {\cos \theta_{x} + \frac{m\lambda }{{p_{x} }}} \right)^{2} - 1} }}$$

where $cos\theta_{x} = k_{x} /k$ and $cos\theta_{y} = k_{y} /k.$ In the receiving/absorbing mode, the MA must be suitably terminated by a resistive load (A piece of AgNw lossy sheet is utilized in this paper) to harvest the maximum power impinging on the array. It should be noted that another lossy material in the THz spectrum, such as graphene^9^, could also be utilized to dissipate the trapped power. The reciprocity condition ensures that the reflectivity seen by a plane wave looking into the MA array (in the absorbing mode) is equivalent to the reflectivity seen looking into the terminal of the dipoles (in the transmit mode)

8 $${\text{S}}_{11} \left( {\theta ,\varphi } \right) = \frac{{Z_{element} \left( {\theta ,\varphi } \right) - R_{L} }}{{Z_{element} \left( {\theta ,\varphi } \right) + R_{L} }}$$

in which $R_{L}$ denotes the terminated/source impedance. The absorption spectrum of the dipole MA can be calculated from A = 1 − R − T, in which R =|S_11_|^2^ and T =|S_12_|^2^ are the reflectance and transmittance of the array, respectively. Nevertheless, since the gold ground plane thickness is much larger than THz skin-depth, we have no transmission (*T* = *0*) across the frequency band of interest. The related parameters of the designed MA are $p_{x} = p_{y} = 0.5\lambda$, $l = 0.48\lambda$, $w = 0.02\lambda$, $h_{1} = 0.25 \lambda$, and R_L_ = 50 Ω where $\lambda$ denotes the operating wavelength corresponding to f = 2 THz. A MATLAB program was prepared to implement the input (scan) impedance of the MA array whose accuracy with the involved multiple harmonics is compared with the benchmark full-wave numerical solution (CST commercial software). An adequate number of Floquet harmonics, at least including m, n $\in \left\{ {0, \pm 1, \pm 2, \pm 3, \ldots , \pm 10} \right\}$, is considered here to reach acceptable accuracy in the analysis, as the other harmonics were found to have negligible contributions. On the other hand, in the full-wave modelling of the proposed MA, periodic boundary conditions are applied in both the x- and y-directions while the Floquet port illuminates the structure along the z-direction. Figures 2a, b compare, respectively, the analytical and numerical results of absorptivity for the employed MA in both E- (yOz plane) and H-planes (xOz plane). As the inset of these figures show, an acceptable agreement is noticed between the reference solutions and the analytical results. The E-plane absorptivity deteriorates at the boresight direction since the scan impedance of the array is high in this angular regime (about 150 Ω). Besides, even though the absorptivity variation of the array is slightly smoother in the E-plane, the angular dispersion is not weak in both planes. This observation can be attributed to the mutual coupling between the elements where the reactive component of the input impedance for the employed MA increases with the scan angle^49^. Therefore, due to the air-absorber impedance mismatch, the MA array of Fig. 2 exhibits an angle-sensitive performance in both principal planes, where reflectivity approaches 100% near the grazing angle.

## The near-field interactions between MA array and uniaxial superstrate layer

**Figure 3:**
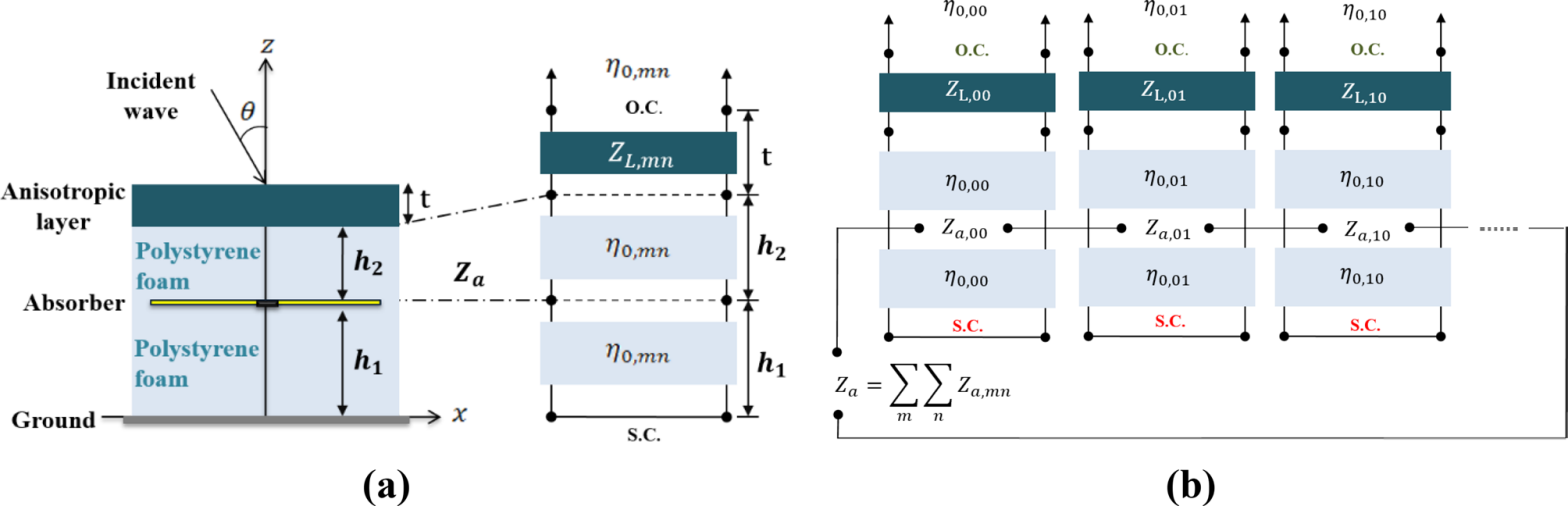
(**a**) The cross-sectional schematic of the unit cell of the proposed SLMA and the equivalent circuit model, including a certain number of cascaded lines. The near-filed interactions between the MA and the superstrate layer can be theoretically represented by (**b**) uncoupled transmission line networks illustrating the series summation of individual Floquet impedances.

For optimal MA performance upon illumination by oblique incidences, it is highly desirable to counteract the strong angular dependency of the input impedance of the dipole MA with an auxiliary anisotropic layer located at a certain distance, *h*_2_, above the array (see Fig. 3a). Assuming an anisotropic superstrate layer characterized by diagonal (uniaxial) magnetic and dielectric tensors^56^

9 $$\overline{\overline{\varepsilon }}_{2} = \left[ {\begin{array}{*{20}c} {\varepsilon_{2x} } & 0 & 0 \\ 0 & {\varepsilon_{2y} } & 0 \\ 0 & 0 & {\varepsilon_{2z} } \\ \end{array} } \right]$$

10 $$\overline{\overline{\mu }}_{2} = \left[ {\begin{array}{*{20}c} {\mu_{2x} } & 0 & 0 \\ 0 & {\mu_{2y} } & 0 \\ 0 & 0 & {\mu_{2z} } \\ \end{array} } \right]$$

will substantially simplify the synthesis and enhance the feasibility of our design. Since the evanescent Floquet harmonics in the vicinity of the array and the superstrate layer can highly interact with the discontinuity caused by the anisotropic layer, the resultant effect on the scan impedance of the MA becomes greater as the superstrate approaches the array. Inspired by the form of Eq. (6), a network of uncoupled transmission lines can be served to take into account such near-field interactions between the MA elements and the anisotropic superstrate layer (with the thickness *t* above the array). Keeping the reciprocity theorem in mind, the fields of the dipole array (in the radiation mode) can be expanded with a set of distinct Floquet harmonics, each of which can be represented by a simple equivalent transmission line model. It is for the first time that the role of evanescent Floquet harmonics is theoretically assessed when designing MAs augmented by auxiliary anisotropic layers. Here, considering different media above the dipole elements, a certain number of clustered cascaded lines with different characteristics describe each harmonic (Fig. 3b). According to the developed semi-analytical relations for MA (array of dipoles terminated by the lossy AgNw sheet), it can be electromagnetically incorporated before the uncoupled transmission lines modelling the overlaying media at each Floquet harmonic. Upon being affected by the anisotropic impedance-matching layer, the new scan impedance at the input aperture discontinuity of the array can be calculated as a series summation of the individual scan impedances belonging to each Floquet harmonic (Fig. 3b). The latter case can be simply computed based on several transmission line impedance transformations to move the impedance at the boundary $z = h_{1} + h_{2} + {\text{t}}$ to that of $z = h_{1}$. Here, we will treat the problem in separate cases of TM and TE polarizations where only the $\varepsilon_{y}$, $\varepsilon_{z}$, and $\mu_{x}$ components matter for TM polarization, while only the $\varepsilon_{x}$, $\mu_{y}$, and $\mu_{z}$ components play the matching role for TE polarization.

In this fashion, the EM properties of the anisotropic layer, i.e. the wave impedance and normal component of the wave vector, can be written as^47^

11a $$Z_{L,TE} = \frac{{\omega \mu_{0} \mu_{x} }}{{k_{z,TE} }}$$

11b $$Z_{L,TM} = \frac{{k_{z,TM} }}{{\omega \varepsilon_{0} \varepsilon_{x} }}$$

11c $$k_{z,TE} = \sqrt {k^{2} \mu_{x} \varepsilon_{y} - \left( {k_{xm}^{2} + k_{yn}^{2} } \right)\frac{{\mu_{x} }}{{\mu_{z} }}}$$

11d $$k_{z,TM} = \sqrt {k^{2} \mu_{y} \varepsilon_{x} - \left( {k_{xm}^{2} + k_{yn}^{2} } \right)\frac{{\varepsilon_{x} }}{{\varepsilon_{z} }}}$$

Moreover, the addition of the anisotropic layer also re-defines Eq. (6) considering the multiple reflections occurring between the nearby interfaces^49^

12 $$Z_{a,modified} = \frac{2\eta }{{\pi^{2} }} \frac{{w^{2} }}{{p_{x} p_{y} }}\sum\limits_{m = - \infty }^{\infty } {\sum\limits_{n = - \infty }^{\infty } {F_{m}^{2} G_{n}^{2} \left( {H_{mn}^{TE} \eta_{mn}^{TE} \gamma_{mn}^{TE} + H_{mn}^{TM} \eta_{mn}^{TM} \gamma_{mn}^{TM} } \right)} }$$

in which

13a $$H_{mn}^{TE} = \frac{{k_{xm}^{2} }}{{k_{xm}^{2} + k_{yn}^{2} }}$$

13b $$H_{mn}^{TM} = \frac{{k_{yn}^{2} }}{{k_{xm}^{2} + k_{yn}^{2} }}$$

13c $$\gamma_{mn}^{TE/TM} = \left( {1 + {\Gamma }_{out,mn}^{TE/TM} exp\left( { - j4 \pi \nu_{mn} h_{2} } \right)} \right) \times \frac{{1 - exp\left( { - j4 \pi \nu_{mn} h_{1} } \right)}}{{1 + {\Gamma }_{out,mn}^{TE/TM} exp\left( { - j4 \pi \nu_{mn} \left( {h_{1} + h_{2} } \right)} \right)}}$$

14 $${\Gamma }_{out,mn}^{TE/TM} = \frac{{Z_{b}^{TE/TM} - Z_{0}^{TE/TM} }}{{Z_{b}^{TE/TM} + Z_{0}^{TE/TM} }}$$

The impedances for each Floquet harmonic, $\eta_{mn}^{TE}$ and $\eta_{mn}^{TM}$, are given by $k/\nu_{mn}$ and $\nu_{mn} /k$, respectively, and ${\Gamma }_{out,mn}^{TE/TM}$ stands for the reflection observed looking into the anisotropic layer. Besides, $Z_{b}^{TE/TM}$ represents the transferred impedance of the free space medium at the boundary ($z = h_{1}$ + $h_{2}$ + t) to that of $z = h_{1}$ + $h_{2}$. Hereafter, Eq. (12) can serve to compute the modified scan impedance and the absorptivity of the proposed SLMA, i.e. the new absorber architecture augmented by the uniaxial anisotropic superstrate. As the dimensions of the proposed meta-atom are comparable with the wavelength (p = 0.5λ), care should be taken about the presence of high-order Floquet harmonics (diffraction modes) in the calculation of absorptivity values, especially in the oblique incidence situation. A comprehensive discussion is given in Supplementary Information A. In this case, the absorptivity of the proposed structure can be computed based on:

15 $$A\left( \omega \right) = 1 - \mathop \sum \limits_{m} \mathop \sum \limits_{n} \left| {S_{11}^{{TE\left( {TM} \right)/TE\left( {TM} \right)}} \left( {m,n,\omega } \right)} \right|^{2} - \mathop \sum \limits_{m} \mathop \sum \limits_{n} \left| {S_{11}^{{TE\left( {TM} \right)/TM\left( {TE} \right)}} \left( {m,n,\omega } \right)} \right|^{2}$$

in which, the second term denotes the co-pol, and the third one refers to the cross-coupling impacts of the high-order Floquet harmonics. Also, parameters *m* and *n* denote the index of each diffraction mode. All results presented throughout this paper have been computed considering a truncated number of diffraction modes.

In the first step, we prepared a constrained optimization to seek optimum parameters revealing the best wide-angle absorption performances among all the possible solutions that may, however, be arduous to be realized. The magneto-electric parameters of the anisotropic matching layer are allowed to be freely varied within intervals, excluding extreme values. The constraints used in the optimization at f = 2 THz are tabulated in Table 1. This optimum solution is utilized only as a benchmark to demonstrate the wide-angle impedance matching caused by the anisotropic superstrate.

**Table 1:** The optimum parameters of the magneto-dielectric anisotropic superstrate layer obtained by solving the constrained optimization problem at f = 2 THz.

Designed properties	Parameter intervals	Values
$\varepsilon_{x}$	[0–10]	5
$\varepsilon_{y}$	[0–10]	5.63
$\varepsilon_{z}$	[0–10]	1.04
$\mu_{x}$	[0–10]	0.416
$\mu_{y}$	[0–10]	0.2
$\mu_{z}$	[0–10]	0.247
$t$	[0.05–0.15] × *λ*	$0.04 \times \lambda$
$h_{2}$	[0.05–0.15] × *λ*	$0.078 \times \lambda$

**Figure 4:**
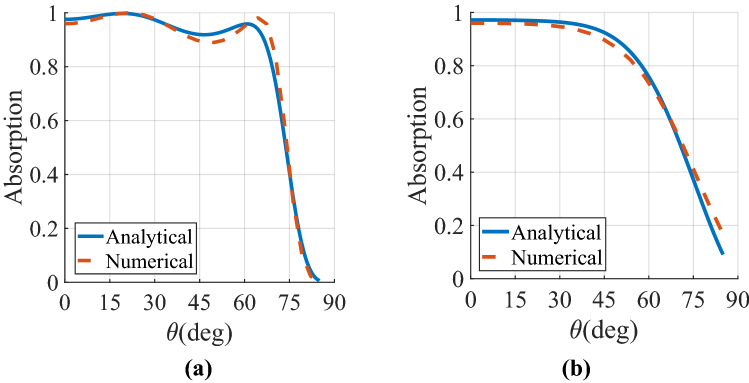
Comparison between the analytical and numerical absorptivity at the specular angle of the designed SLMA with the bulk superstrate layer characterized in Table 1 for incident angles θ ranging from 0° to 90° in (**a**) $\varphi = 0$ and (**b**) $\varphi = 90$ planes. The MA has the parameters of p_x_ = p_y_ = 0.5λ, *l* = 0.48λ, w = 0.02λ, h_1_ = 0.25 λ, and R_L_ = 50 Ω where λ denotes the operating wavelength corresponding to f = 2 THz.

For a set of 300 randomly generated starting points, the local minima of the cost function are repeatedly sought. Figure 4a, b plot the resultant absorption curves versus the angle of incidence in both E- and H-planes, respectively. Evidently, the theoretical and numerical results perfectly overlap each other, confirming the validity of our proposal for analyzing the proposed SLMA. Consequently, the analysis yields exact results, notwithstanding the simplifying assumptions made in the construction of the model. Compared to Fig. 2, the results have been significantly improved in terms of both efficiency and angular-stability in the two principal planes. The new architecture of MA (i.e. the designed SLMA) depicts strong absorptivity above 0.8 upon illumination by all incident wave angles up to 72° for E-plane and 65° for H-plane.

## Realizing the wide-angle SLMA

**Figure 5:**
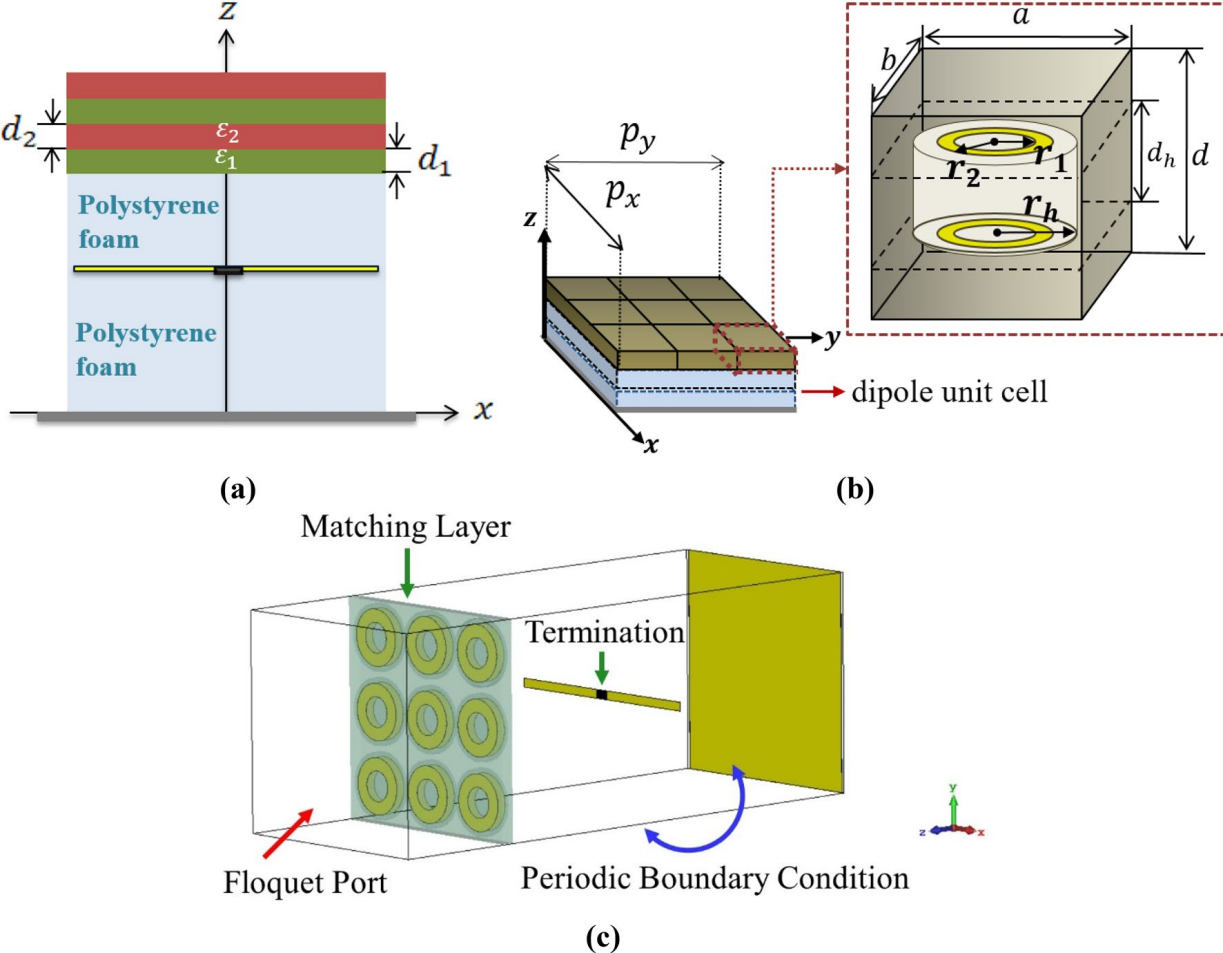
The schematic view of the proposed SLMA structures augmented by (**a**) the electric-only multilayer dielectric superstrate and (**b**) magneto-electric Huygens' metamaterial superstrate. (**c**) The full-wave simulation setup for the designed SLMA with magneto-electric Huygens' metamaterial superstrate.

The anisotropic parameters used for the results of Fig. 4a, b are best-case solutions assuming that all dielectrics with arbitrary EM parameters are available, which is not true in real-life scenarios. To circumvent this unrealistic assumption and due to the importance of practical considerations, this study discloses two distinct accomplishable proposals, i.e. electric-only and magneto-electric uniaxial superstrate layers, to implement the designed SLMA. Figure 5a, b illustrate the topology of two configurations of SLMA whereby the angular performance of the superstrate layers is computationally verified. To realize the electric-only uniaxial layer, we adopt the superstrate by periodically stacking two commercially available homogeneous materials with different permitivities and thicknesses of $(\varepsilon_{r1} ,\varepsilon_{r2} )$ and $\left( {d_{1} , d_{2} } \right)$ along the perpendicular direction (see Fig. 5a). The equivalent tensor parameters of the multilayer dielectric system are retrieved in the absence of the array of dipole resonators. This is a common practice in analyzing the anisotropic superstrates loading a periodic array^47^. Based on the effective medium theory^57,58^, the equivalent tensor components of the dielectric permittivity can be derived as

16a $$\varepsilon_{x} = \varepsilon_{y} = \frac{{\varepsilon_{r1} d_{1} + \varepsilon_{r2} d_{2} }}{{d_{1} + d_{2} }}$$

16b $$\varepsilon_{z} = \frac{{\left( {d_{1} + d_{2} } \right)\varepsilon_{r1} \varepsilon_{r2} }}{{\varepsilon_{r1} d_{2} + \varepsilon_{r2} d_{1} }}$$

On the other hand, to realize the magneto-electric anisotropic layer, we employ two parallel gold rings embedded in a host dielectric medium (polyethylene-high density with the refractive index of n = 1.54^59^), separated by an air hole. The Huygens' metamaterial will strongly interact with both electric and magnetic incident fields, and its working principle can be individually evaluated for the tangential and normal components. The magnetic and electric parameters can be controlled by tuning the geometric parameters in a coupled manner^60^. In this case, the simple volumetric averaging of Eqs. (16a, b) is no longer applicable, and a more complicated approaches should be employed. Kramers–Kronig limitation is applicable to any material parameter intended to have a physical sense of a generalized susceptibility. Care should be taken that the selected system of describing parameters may fail to obey the relevant restrictions when its parameters, such as frequency-dispersive permittivity and permeability, are not sufficient to describe the actual response of the structure. In this case, a more extended theoretical description is required, taking spatial dispersion into account^61–67^.

## Numerical simulations

**Figure 6:**
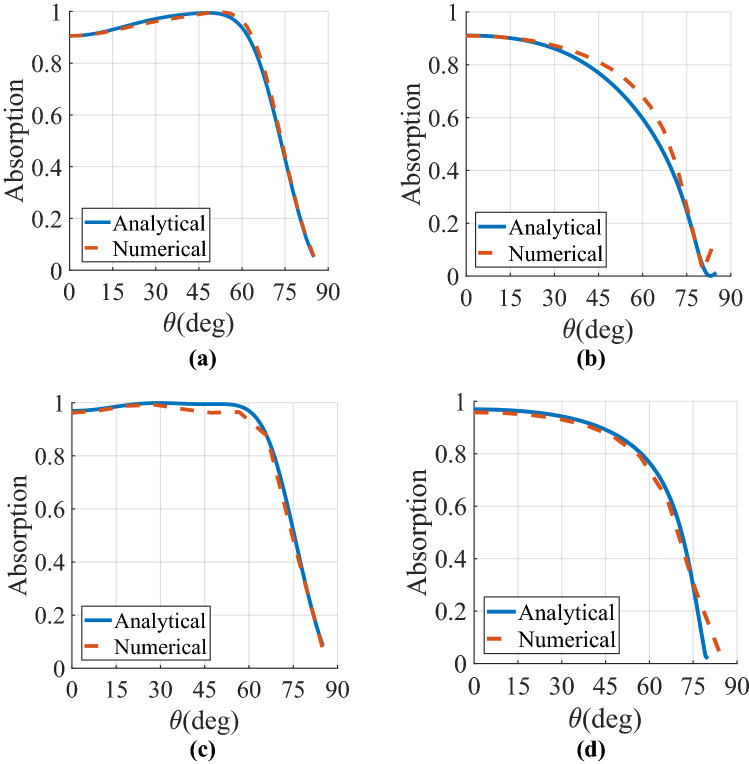
Comparison between the analytical and numerical absorptivity values of the proposed SLMA at the specular angle with the (**a**), (**b**) dielectric-only and (**c**, **d**) magneto-electric anisotropic superstrate (Huygens' metamaterial) layer for incident angles θ ranging from 0° to 90° in (**a**, **c**) $\varphi = 0$ and (**b**, **d**)$\varphi = 90$ planes. The MA has the parameters of p_x_ = p_y_ = 0.5λ, *l* = 0.48λ, w = 0.02λ, h_1_ = 0.25λ, and R_L_ = 50 Ω where λ denotes the operating wavelength corresponding to f = 2 THz.

**Figure 7:**
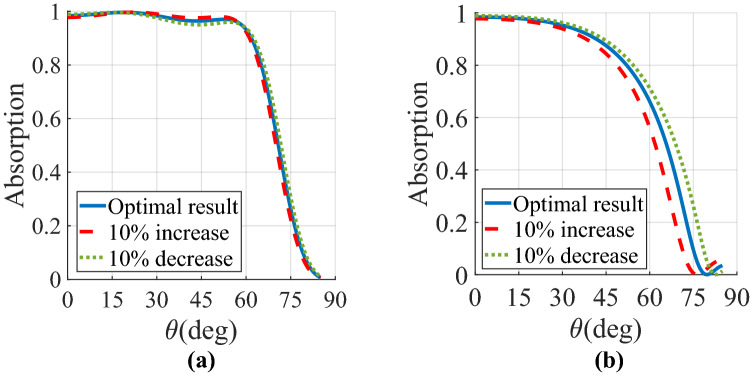
Performance assessment of the Huygens’ metamaterial anisotropic superstrate on the absorptivity of the proposed SLMA at f = 2 THz and (**a**) $\varphi = 0\user2{ }$ and (**b**) $\varphi = 90$ when the constitutive parameters experience a 10% tolerance.

**Figure 8:**
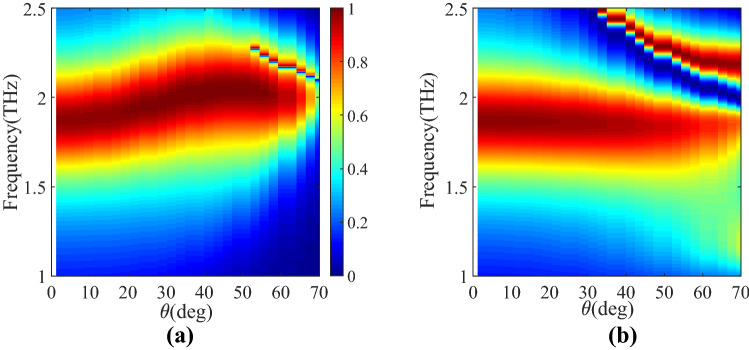
The absorptivity spectrum of the designed MA loaded with the optimized magneto-electric anisotropic superstrate (realized by Huygens' metamaterial) as a function of frequency and the incident wave angle in (**a**) $\varphi = 0$ and (**b**) $\varphi = 90$ planes.

In this section, we aim to investigate the broad angular performance (or weak angular dispersion) of the SLMA realized by the multilayer dielectrics and the Huygens' metamaterial over the aperture plane of the MA. It should be noted that, although the Huygens' metamaterial is established to realize the best parameters given in Table 1, these parameters cannot be implemented by the multilayer dielectric system as it does not provide any magnetic response. Therefore, a new optimization procedure was employed, and the optimum dielectric tensor parameters were accordingly achieved. The best parameters were searched within a feasible database of THz materials available in practice, i.e., among the anisotropic parameters which are neither extremely high nor near-zero nor negative, as strong frequency dispersion and non-negligible losses will appear in these regimes. Finally, a specifically designed stack of Al_2_O_3_ and PMMA layers was chosen to realize the required anisotropy of the superstrate. The optimum parameters are given in Table 2. Moreover, to find the optimal geometries of the Huygens' metamaterial mimicking the parameters of Table 1, extensive parametric sweeps were performed at the operating frequency of 2 THz. Being located far from the structure resonance, the proposed meta-atom almost treats in a non-dispersive routine in frequency. The main reason can be attributed to the fact that the magnetic polarization of a subwavelength metal ring has a flat frequency trend^47^. This can be mainly attributed to the fact that the metamaterial unit has no vias and operates far from any structural resonance. For further validation, the full-wave simulation results, as well as the theoretical predictions for the best solution of both SLMA designs, are displayed in Fig. 6a–d. The configuration of the simulation setup is shown in Fig. 5c in which the realized superstrate (double gold ring structure) and the dipole array are considered in a single simulation system, and the absorptivity of the whole system is extracted. As can be seen, nine sub-wavelength MM elements are located in each cell of the designed SLMA. The comparison depicts an almost perfect agreement between full-wave and semi-analytical results on the E- and H-planes, respectively. The results demonstrate that the magneto-electric Huygens' layer modifies the angular performances of the dipole MA array almost in a similar fashion to the multilayer superstrate for the E-plane, whereas due to providing both electric and magnetic behaviors, better response in the absorption has been observed on the H-plane. In this plane, the electric-only multilayer superstrate functions relatively well at lower scan angles, but its performance degrades in middle and higher scan angles. Overall, via the proposed designs, the absorbed power of the designed SLMA structures is dramatically increased in both E- and H-planes when excited by a broad range of oblique incidences, a great capability that has not been analytically reported yet. Approximately, a 70% portion of the absorbed power is observed in the E-plane for oblique incidences up to 80° at the operating frequency. It is clear that our design is normalized to the wavelength and hence applicable to any range of the EM spectrum. However, one should be very careful with the model used for the metallization at THz as the nominal conductivity does not fit properly. Therefore, the practical aspects of the design can be further considered by reducing the nominal DC conductivity by order of magnitude to account for extra loss introduced by roughness. The corresponding results can be found in Supplementary Information B. To evaluate the sensitivity of the SLMA absorption (realized by Huygens' metamaterial) upon deviations in the anisotropic parameters of the employed superstrate (possible fabrication tolerances), we present the results in Fig. 7a, b for the performance of the optimized SLMA of Fig. 5b when the magnetic or/and electric tensor parameters deviate by 10% from the optimized solutions. Indeed, a 10% variation is considered for all the electric and magnetic tensor parameters. As the inset of this figure illustrates, the design is almost robust against the possible fabrication tolerances which may occur during the manufacturing processes, especially in the E-plane. Besides, since the frequency bandwidth is also a critical figure of merit in most THz systems, it is important to consider the implications of the anisotropic layers, whose particles' individual response may experience a rapid spectral change on the bandwidth of the dipole MA array. As discussed before, for the employed subwavelength particle realizing the Huygens' anisotropic metamaterial, the resonance frequency is far enough from the working frequency of 2 THz, so that the SLMA operates in a less frequency dispersive and less lossy regime of the anisotropic layer. In order to clearly reveal the spectrally-selective and wide-angle absorption properties of the proposed SLMA (realized by Huygens' metamaterial), we illustrate the absorption spectra at different angles of incidence in Fig. 8a, b. On the one hand, the value of the absorption peak is 0.98 for normal incidence. With increasing the incident wave angle, the value of the absorption peak remains interestingly high (around unitary) in both E- and H-planes. However, there is a slight frequency shift with an increase of *θ*, thus exhibiting minimal dispersion within the operating bandwidth. Obviously, the frequency bandwidth of the SLMA, which is almost dictated by the dipole elements, is not deemed as the main topic in this paper; nevertheless, the other engineered shapes for the primary MA can be selected to broaden the absorption band or for dual-polarized operation. In these cases, the EM field excited by the basic array can still be taken into account with semi-analytical models, when regular geometries such as rectangular and circular apertures and printed slots are involved. In addition, resorting to full-wave simulations is an alternative approach, which allows the analysis of arbitrarily shaped MA units^68^. Indeed, the proposals given in this paper do not necessarily postulate the primary knowledge about the analytical investigation of the MA element, as they can be numerically applied to any type of MA geometry.

**Table 2:** The optimum parameters of the dielectric anisotropic superstrate layer obtained by solving the constrained optimization problem.

Designed properties	Values	Material
$\varepsilon_{r1}$	9.4	Al_2_O_3_ (tan $\delta$ = 0.0004)
$\varepsilon_{r2}$	2.53	PMMA (tan $\delta$ = 0.006)
$d_{1}$	1 μm	
$d_{2}$	8 μm

## Discussion

A wide-angle and spectrally-selective THz SLMA based on a fully analytical framework involving the near-field interactions between the Floquet harmonics and the superstrate layer has been presented in this paper for the first time. Using transmission line networks and relying on the reciprocity theorem, two designs based on an array of dipoles armed with uniaxial superstrate were sketched and analyzed. The multilayer dielectric system and the Huygens' metamaterial are separately utilized for realizing the electric-only and magneto-electric anisotropic superstrates, respectively. The comparison of the full-wave simulations and theoretical results for both designs revealed perfect similarity, verifying the proposed analytical scheme. It was demonstrated that, when suitably selecting the anisotropic parameters, the efficiency of MA could be substantially improved for a wide range of incident angles, including near grazing angles. As critical figures of merit, the effects of the frequency variation and the possible fabrication tolerances were investigated for the proposed SLMA. The proposals discussed in this paper do not necessarily postulate the primary knowledge about the analytical investigation of the MA element, as they can be numerically applied to any type of MA geometry when other features like broader bandwidths are highly demanded. Overall, the novelty of this paper was not only the design of a wide-angle THz MA but the proposal of a fully theoretical algorithm to expand the angular operation of a pre-designed absorber, regardless of its operating frequency. The main contributions of this paper are: (1) for the first time, our study accurately explored the interaction between the superstrate structure and the dipole array by incorporating the higher-order Floquet modes in the transmission line model; (2) the design is independent of the center frequency and can be re-performed for all spectra from microwave to visible; (3) the proposals given in this paper do not necessarily postulate the primary knowledge about the analytical investigation of the MA element, as they can be numerically applied to any types of MA geometry, e.g. to broaden the absorption band. The wide-angle property of the proposed SLMA would be very promising as an absorbing element for various applications such as THz imaging. Other appealing features can also be envisioned for the proposed structure, such as tunable functionality provided by the graphene^69–72^ material.

## Supplementary information

Supplementary information
