# Supplementary material for: A theoretical investigation on reciprocity-inspired wide-angle spectrally-selective THz absorbers augmented by anisotropic metamaterials
Source: Sci Rep. 2020 Jun 25;10:10396. doi: 10.1038/s41598-020-67399-3 (PMC7316824; doi:10.1038/s41598-020-67399-3)
Supplement: Supplementary file 1 — Supplementary information [file 41598_2020_67399_MOESM1_ESM.docx]

Supplementary Information for

**A Theoretical Investigation on Reciprocity-Inspired Wide-Angle Spectrally-Selective THz Absorbers Augmented By Anisotropic Huygens' Metamaterials**

**Mansoureh Mohammadi**^1,2,+^**, Hamid Rajabalipanah**^1,2,+^**, and Ali Abdolali**^1,2,*^

^1^ Department of Electrical Engineering, Iran University of Science and Technology, Tehran, 1684613114, Iran

^2^ Applied Electromagnetic Laboratory, School of Electrical Engineering, Iran University of Science and Technology, Tehran, 1684613114, Iran

*abdolali@iust.ac.ir

^+^these authors contributed equally to this work

**The Supplementary file includes:**

The detailed explanations of 1) Deteriorating effects of diffraction modes; 2) Sensitivity analysis for conductivity values.

**Supplementary Appendix A
(Deteriorating effects of diffraction modes)**

The proposed metamaterial absorber is characterized by a periodicity about half-wavelength (at the operating frequency) which would cause excitation of a certain number of high-order Floquet harmonics, especially for oblique illuminations. In this way, the energy may be efficiently spread towards high order harmonics in the same frequency. We will prove that our selection on the structure dimension (p=0.5λ) avoids propagation of higher order modes and there should be no concern about this issue.

When an electromagnetic wave impinges on an interface, it can be transmitted, reflected or absorbed. In the most general state, the direction of the reflected and transmitted waves obeys the generalized Snell's law [S1]. However, when the interface is a periodic surface with an inter-element distance about half-wavelength, the interference phenomena make scattered waves to be deflected into multiple directions known as diffraction orders or Floquet harmonics [S2]-[S5]. The propagation directions are merely determined by the geometry of the lattice on which the employed unit cells are arranged. In a one-dimensional case, the non-specular scattering directions can be determined based on the basic theory of diffraction gratings [S6]. For a bidirectional periodic array, the additional reflected components are characterized by the following in-plane wave-vector components

(S1)

where, , , and $k_{z}=\sqrt{k_{0}^{2}-k_{x}^{2}-k_{y}^{2}}$, p_x_ and p_y_ indicate the inter-element spacing, (m, n) represent the indexes of the Floquet harmonics and finally, (𝜃, 𝜑) are azimuth and elevation angles of the incident wave. It is evident from the above relations that a certain number of higher order Floquet modes may propagate when the periodicity of absorber is about the half-wavelength upon illuminating by oblique incidences. In this case, it could happen that in the simulation one gets low reflection in the zero-order mode because some of the energy goes to a higher order mode rather than being totally absorbed. Although the high-order Floquet harmonics can be potentially excited, the excitation level for each of them strongly depends on the shape, geometrical dimensions, and constitutive materials of the employed unit cell [S7]. Therefore, the presence of high-order harmonics does not necessarily deteriorates the absorption results.

Theoretically speaking, the total scattered field can be reduced to a summation of harmonic contributions:

$\left\{\begin{matrix} E_{ref}=\sum_{i=-\infty}^{\infty} R_{i}\exp\left(j \bar{k}_{ i} .\bar{r}\right) \\ P_{ref}\propto\left|E_{ref}\right|^{2} \end{matrix}\right.$ (S2)

where, k_i_ is the wave vector of the i^th^ harmonic, r indicates the propagation direction and Ri denotes the reflection coefficient of the i^th^ harmonic towards a specific direction. The energy carried by a specific harmonic is related to the diffraction efficiency. Based on Eq. (S1), the Floquet harmonics will appear at lower frequencies as the angle of incidence becomes greater.

Now, we intend to evaluate the cut-off frequencies (in which k_z_=0) of several high order Floquet modes (m&n=0, ±1, ±2) scattered by the proposed metamaterial absorber for different incident wave angles. The corresponding results are plotted in Figure Supplementary S1. As can be seen, owing to the moderate size of the designed unit cell, the modes m=−1, n=0 (φ_inc_=0) and m=0, n=−1 (φ_inc_=90°) are only propagative near f=2THz, and the other spatial harmonics are evanescent and do not contribute to the total absorption which is a far-field measure.

Besides, the strength of these two harmonics should be checked by plotting their reflection coefficient (cross-coupling, R_i_) in order to determine how much the power will be diffracted (scattered) and how much else will be absorbed. Figures Supplementary S2a, b illustrate the reflectance of (m=1, n=0) and (m=0, n=1) Floquet modes for different incident wave angles, respectively, to numerically prove that a very small amount of the power is diffracted to the other non-specular directions. From Figures Supplementary S2a, b, one can conclude that the strength of high-order Floquet modes is actually weak (|R_i_|^2^<0.0002) at f=2THz and in another sense, a negligible portion of the incident energy (less than 0.02%) is coupled to the diffraction (scattering) modes.

We have considered several high order modes during our simulations to extract the absorptivity of structure, however, the authors could safely ignore them in their simulations as the energy coupled to these high order modes were not strong enough. In this case, the absorption formula should be modified to include the possible effects of such diffraction modes:

$A\left(\omega\right)=1-\sum_{n} \sum_{m} \left|S_{11}^{TE(TM)/TE(TM)}(m,n,\omega)\right|^{2}-\sum_{n} \sum_{m} \left|S_{11}^{TE(TM)/TM(TE)}(m,n,\omega)\right|^{2}$ (S3)

|  |  |
| --- | --- |
| (a) | (b) |
|  |  |
| (c) | (d) |
|  |  |
| (e) | (f) |
|  |  |
| (g) | (h) |
|  |  |
| (i) | (j) |
|  |  |
| (k) | (l) |
|  |  |
| (m) |  |

**Supplementary Figure S1.** (a)-(m) The cut-off frequencies of the first high-order modes for the designed metamaterial-based absorber versus incident wave angles. The index of each mode is indicated above each figure.

in which, the second term denotes the co-pol coupling effects and the third one refers to the cross-coupling impacts.

Moreover, we neglect the transmission of the incident wave through the designed absorber since a metal plate terminates the structure. Albeit, as discussed above, the (m=−1, n=0) and (m=0, n=−1) harmonics are only effective in our design (see Supplementary Figure S3). However, the absorptivity of the proposed metamaterial absorber illustrated in Figures 4,7,8, and 9 of the original manuscript was computed by considering the reflection coefficients pertaining to the high-order modes (diffraction modes) where the above summation was truncated by |m|=2, |n|=2.

|  |  |
| --- | --- |
| (a) | (b) |
| **Supplementary Figure S2.** The reflectance spectra of the designed metamaterial-based absorber for two high-order harmonics of (a) m=1, n=0 and (b) m=0, n=1. | |

As can be seen, the high order floquet modes is not strong enough to significantly deteriorate the absorption efficiency and the angular behavior of the designed metamaterial absorber (The higher order modes |m|≥3 and |n|≥3 are also considered by the authors and did not deteriorate the results. So, for the sake of briefness, the corresponding results are not given here).

|  |  |
| --- | --- |
| (a) | (b) |
| **Supplementary Figure S3.** The angular spectra of the reflectance of the designed metamaterial-based absorber for two high-order harmonics of (a) m=−1, n=0 and (b) m=0, n=−1. | |

**Supplementary Appendix B
(Sensitivity analysis for conductivity values)**

The design in this paper is normalized to the wavelength and hence applicable to any range of the electromagnetic spectrum. However, we have focused on terahertz frequencies as designing wide-angle metamaterial absorbers with rigorous analytical formulations has not been presented so far. In this frequency regime, performing a sensitivity analysis for conductivity values of metals is necessary through reducing the nominal DC conductivity by an order of magnitude to account for extra loss introduced by roughness. The corresponding results are given in Supplementary Figure S4. As can be observed, the proposed absorber retains its good performance with a negligible change in the efficiency for different conductivity values ranging from σ=5×10^6^ S/m to σ=4.5×10^7^ S/m.

|  |  |
| --- | --- |
| (a) | (b) |
| **Supplementary Figure S4.** The absorption spectra of the designed SLMA (Figure 5b of the manuscript) for different conductivity values used for the metallization. | |

**References**

1. Yu, Nanfang, Patrice Genevet, Mikhail A. Kats, Francesco Aieta, Jean-Philippe Tetienne, Federico Capasso, and Zeno Gaburro. "Light propagation with phase discontinuities: generalized laws of reflection and refraction." science 334, no. 6054 (2011): 333-337.
2. Palmer, Christopher A., and Erwin G. Loewen. Diffraction grating handbook. New York: Newport Corporation, 2005.
3. Munk, Benedikt A. Frequency selective surfaces: theory and design. Vol. 29. New York: John Wiley, 2000.
4. Cope, Thomas D. "The Rittenhouse diffraction grating." Journal of the Franklin Institute 214.1 (1932): 99-104.
5. Orazbayev, Bakhtiyar, Pablo Rodríguez-Ulibarri, and Miguel Beruete. "Wideband backscattering reduction at terahertz using compound reflection grating." Optics Express 25.19 (2017): 22905-22910.
6. Gaylord, T. K., and M. G. Moharam. "Planar dielectric grating diffraction theories." Applied Physics B 28.1 (1982): 1-14.
7. Costa, Filippo, Agostino Monorchio, and Giuliano Manara. "Wideband scattering diffusion by using diffraction of periodic surfaces and optimized unit cell geometries." Scientific reports6 (2016): 25458
